# How to keep up with the analysis of classic and emerging neurotoxins: Age-resolved fitness tests in the animal model *Caenorhabditis elegans* - a step-by-step protocol

**DOI:** 10.17179/excli2021-4626

**Published:** 2022-01-31

**Authors:** Indra Hering, Dang Tri Le, Anna von Mikecz

**Affiliations:** 1 IUF - Leibniz Research Institute for Environmental Medicine

**Keywords:** aging, climate change, exposome, neurotoxicity, pollution

## Abstract

The global chemical inventory includes neurotoxins that are mostly interrogated concerning the biological response in developing organisms. Effects of pollutants on adults receive less attention, although vulnerabilities can be expected throughout the entire life span in young, middle-aged and old individuals. We use the animal model *Caenorhabditis elegans* to systematically quantify neurological outcomes by application of an age-resolved method. Adult hermaphrodite worms were exposed to pollutants or non-chemical stressors such as temperature in liquid culture on microtiter plates and locomotion fitness was analyzed in a whole-life approach. Cultivation at 15, 20 or 25 °C showed that worms held at 15 °C displayed an enhanced level of fitness concerning swimming movements until middle age (11-days-old) and then a decline. In contrast, *C. elegans* cultivated at ≥ 20 °C continually reduced their swimming movements with increasing age. Here, we provide a step-by-step protocol to investigate the health span of adult *C. elegans* that may serve as a platform for automation and data collection. Consistent with this, more neurotoxins can be investigated with respect to vulnerable age-groups as well as contributing non-chemical environmental factors such as temperature.

## Introduction

Pollutants accumulate in the environmental compartments of soil, surface waters, sediments and air. They are distributed worldwide via long range transport and reach remote areas far away from anthropogenic sources (Yuan et al., 2021[27]). As the consequences of pollution in intricate ecosystems such as the arctic or deep sea are largely unknown and climate change adds to environmental stress, a toxicology is needed that integrates investigation of biological responses to chemical and non-chemical stressors (Danovaro et al., 2020[8]; Yuan et al., 2021[27]).

Investigation and evaluation of neurotoxic pollutant effects mostly addresses early development of organisms, while bio-interactions with aging adults receive less attention. However, adverse effects of neurotoxicants also concern aging populations, *e.g.* young, middle-aged and old individuals that are exposed to low doses of chemicals and non-chemical stresses over long time periods. Thus, we propose to focus more on the role of pollutants in aging processes. Neurotoxicology should address comparative analyses of the aging neural system in relation to protein homeostasis, amyloid protein aggregation, neurodegeneration, defective neurotransmission and neuromuscular phenotypes. In a whole-life approach, investigation of pollutant-induced neural aging requires systematic examination of the individuals' neural health span, neurodegeneration and neural function. Notably, such an approach enables the identification of age-specific vulnerabilities.

Investigation of adverse effects of pollutants targeting the neural system in adults requires a suitable animal model with specific key features: (i) a short life span as an adult, (ii) a gene repertoire largely homologous to the human genome including disease genes and complex biochemical pathways, (iii) a neural system that includes neurons as well as glia, and applies the same neurochemistry as humans, (iv) amenability to biochemistry and imaging methods and (v) availability of genetic mutants and gene reporters. Additionally, in order to fulfill the criterion of a realistic target organism of neurotoxicants the animal model should inhabit environmental sinks of pollutants in the wild.

One of the animal models that fulfills all mentioned features is the nematode roundworm *Caenorhabditis elegans*. The wild type hermaphrodite leads a short adult life of 2 - 3 weeks and has a genome containing an estimated 20,470 genes (*C. elegans* sequencing Consortium, 1998[4]) sharing 60-80 % orthologs with the human genome that contains an estimated 19,000 - 20,000 protein-coding genes (International Human Genome Sequencing Consortium, 2001[12]; Kaletta and Hengartner, 2006[13]). The neural system of *C. elegans* comprises exactly 302 neurons with known patterns of synaptic connectivity and neurochemistry (de Bono and Maricq, 2005[9]; Corsi et al., 2015[7]). The transparent nematode is optimally suited for diverse imaging methods, including microscopy of single neurons in reporter worms (Brewer et al., 2019[3]). Additionally, the species *C. elegans* leads a dual life, as model organism in the laboratory and as bacterivore in the wild. Wild nematodes, including *C. elegans*, live in the predicted environmental compartments of neurotoxic pollutants and thus represent realistic target organisms (Wang and Nowack, 2018[25]; von Mikecz, 2018[24]).

In order to observe effects of xenobiotics in an age-resolved manner, we apply the cultivation method that was developed by the Buck laboratory to investigate *C. elegans* longevity and screen for drugs that extend life span (Petrascheck et al., 2007[15]; Solis and Petrascheck, 2011[22]). The quantitative method is based on the co-cultivation of the worms with live bacteria in liquid S-complete medium in 96 well microtiter plates. We observed that in comparison to their counterparts raised on Petri dish solid culture, *C. elegans* live significantly longer in liquid culture, *e.g.* microtiter assays (Piechulek and von Mikecz, 2018[19]). The efficiency of the cultivation method manifested by a significantly increased life span of wild type (N2) *C. elegans* hermaphrodites as well as generally short-lived *daf-16* mutants. Probably due to its analogy with the jellylike, microbe-rich boom habitat on rotting plant material in the wild, this laboratory microhabitat is optimal to study effects of pollutants throughout the entire life span of *C. elegans*. The cultivation format is likewise optimal for whole-life investigation of neurodegeneration and neuromuscular phenotypes such as swimming, since cultivation stress is excluded.

Screening of selected nanomaterials revealed a concentration-dependent reduction of *C. elegans* life span and acceleration of locomotion defects by nano silver (nano Ag) at concentrations ≥ 10 μg/mL, whereas ZnO and 2 types of nano cerium (CeO_2_) showed no adverse biological responses at matching concentrations (1-160 μg/mL; Piechulek and von Mikecz, 2018[19]). Ag nanoparticles (NPs) accelerated the age-associated decline of swimming and increase of uncoordinated movements at concentrations of 10 μg/mL and higher. Notably, this acceleration was significant in 8-day-old worms (p < 0.01) which corroborated that certain pollutants significantly accelerate age-related neuromuscular phenotypes in adult *C. elegans* hermaphrodites at a specific age (Scharf et al., 2013[21]). Middle-aged adult *C. elegans* showed a significant reduction of locomotion after nano Ag NP exposure (10 μg/mL) and young worms (4-day-old) significantly reduced pharyngeal pumping and egg-laying behavior after the exposure to nano silica (Scharf et al., 2013[21]).

Nano Ag induced protein aggregation and axonal beading in the serotonergic hermaphrodite specific neuron (HSN) that was temporally correlated with weakness of the vulva muscles and corresponding egg-laying defects (Scharf et al., 2016[20]; Piechulek and von Mikecz, 2018[19]). After exposure to Ag NPs (> 50 μg/mL) protein aggregates of the reporter worm *tph-1*::DsRed also appeared along axons of the serotonergic neuron ADF. Such beading generally heralds neurodegeneration and impairment of neurotransmission (Scharf et al., 2016[20]; Piechulek and von Mikecz, 2019[18]). The results identified neurodegeneration of serotonergic neurons as an outcome of nano Ag that was observable as early as 26 hours after exposure (Piechulek and von Mikecz, 2018[19]). As respective experiments with *unc-25p*::GFP reporters of GABAergic neurons did not show any biological response to Ag NPs we concluded that (i) different neurons of *C. elegans* possess different sensitivities and (ii) the neurotoxicity of nanomaterials specifically affects certain neurons. Selective neurotoxicity of certain NPs is an interesting finding that deserves further investigation with other pollutants. All investigations with nano silver included the option that dissolution of Ag ions contributed to the observed adverse neural effects. Consistent with this, we showed that AgNO_3_ likewise induced age-dependent neurodegeneration and neuromuscular defects (Piechulek and von Mikecz, 2018[19]). Thus, the concept of life span-resolved studies might not only innovate nanotoxicology, but investigations of pollutants, non-chemical stressors and their mixtures in general.

Toxicology has moved from occupational studies of workplace exposure to population-based environmental investigations. Specific focus is on age-specific vulnerabilities and identification of low dose effects. The concept of the exposome has emerged that aims to develop a comprehensive exposure assessment at the individual level throughout life span and includes the biological response to anthropogenic pollutants (Zhang et al., 2021[29]). In addition to investigation of anthropogenic pollutants, non-chemical stressors such as temperature can be included in the life span-resolved analyses. Consistent with this idea, a step-by-step protocol for a comparative whole-life neuromuscular fitness test at ambient temperatures between 15 and 25 °C is provided below.

The whole-life approach is enabled by a reliable 'laboratory boom habitat' in 96 sample well microtiter plates for analyses of neurotoxicant-effects throughout the entire adult life of the model organism *C. elegans*. Whole-life neurotoxicology allows for the identification of adverse outcomes that are otherwise overlooked in short-term analyses. Adult *C. elegans* develop neuromuscular defects in response to pollutants at a certain age and can thereby be identified as vulnerable target age-groups. For example, the age-associated natural decline of locomotion in wild type worms is accelerated by different pollutants (Piechulek and von Mikecz, 2019[18]). Here, we introduce a life span-resolved fitness test on 96 well and 24 well microtiter plates that enables long-term quantification of the neurobiological response to pollutants.

## Neuromuscular Fitness Test / Step By Step Protocol

### 1 Materials

#### 1.1 Strains

1. *Caenorhabditis elegans* (*C. elegans*) strain Bristol strain N2 (wild type) was received from *Caenorhabditis* Genetics Center (University of Minnesota, MN, USA).
2. *Escherichia coli* (*E. coli*) strain OP50

#### 1.2 Reagents, buffers and solutions

1. Solution A: add 0.5 g cholesterol to 100 ml ethanol (> 99.5 %, absolute) 

2. Solution B: add 11.08 g CaCl_2_ to 100 ml H_2_O, autoclave 

3. Solution C: add 24.65 g MgSO_4_ x 7 H2O to 100 ml H_2_O, autoclave 

4. Solution D: 108.3 g KH_2_PO4, 36 g K_2_HPO_4_ in 1 l H_2_O, autoclave

5. Nematode Growth Medium (NGM) plates: Add 750 ml distilled H_2_O to 15 g BD Bacto Agar, 2.25 g NaCl, 1.9 g BD Bacto Proteose and 3.75 g BD Bacto yeast extract. Autoclave, let the NGM cool down to 55 °C and add the following solutions: 750 µl of solution A, 375 µl of solution B, 750 µl of solution C and 18.75 ml of solution D. Then, pour the NGM into 9 cm Petri dishes under sterile conditions. When the petri dishes are dry, spread 500 µl of an overnight culture of OP50 with an inoculation spreader in the center of the plate^1^ (Stiernagle et al., 1999[23]). 

6. Liquid medium (S-medium): Add 2.93 g NaCl, 0.5 g K_2_PO_4_ and 3 g KH_2_PO_4_ to 500 ml H_2_O and autoclave to sterilize. When the medium has cooled down to 4 °C, add 0.5 ml solution A, 5 ml 1M potassium citrate (pH 6.0), 5 ml trace metals solution, 1.5 ml 1M CaCl_2_ and 1.5 ml 1M MgSO_4_. 205 µl Fungizone Antimycotic (Amphotericin B, stock: stock: 250 µg/ml) are added to prevent the growth of unwanted fungi. Mix the flask by shaking carefully. Next, pour 50 ml of *E. coli* OP50 in a 50 ml sterile conical centrifuge tube and spin for 10 minutes at 2000 g at 4 °C^2^. Discard the supernatant and resuspend the pellet in S-Medium (Stiernagle et al., 1999[23]; Petrascheck et al., 2007[15]).

7. 1 M Potassium citrate pH 6.0: add 20 g citric acid monohydrate and 293.5 g tri-potassium citrate monohydrate to 900 ml H_2_O and shake until the solutes have dissolved. Adjust the volume of the solution to 1 l with H_2_O, adjust the pH value (pH 6.0) and sterilize by autoclaving. 

8. Trace metals solution: add 1.86 g EDTA disodium salt dihydrate, 0.69 g FeSO_4_ x 7 H_2_O, 0.2 g MnCl_2_ x 4 H_2_O, 0.29 g ZnSO_4_ x 7 H_2_O and 0.025 g CuSO_4_ x 5 H_2_O to 900 ml H_2_O and shake until the solutes have dissolved. Adjust the volume of the solution to 1 l with H_2_O and sterilize by autoclaving. Store the solution in the dark. 

9. 1 M CaCl_2_: Dissolve 54 g CaCl_2_ x 6 H_2_O in 150 ml H_2_O. Adjust the volume of the solution to 200 ml with H_2_O and sterilize by autoclaving.

10. 1 M MgSO_4_: Dissolve 120.4 g MgSO_4_ (anhydrous) in 900 ml distilled H_2_O. Adjust the volume of the solution to 1 l with H_2_O and sterilize by autoclaving. 

11. Worm buffer M9: 3 g KH_2_PO_4_, 6 g Na_2_HPO_4_, 0.5 g NaCl and 1 g NH_4_Cl in 1 l H_2_O. Sterilize by autoclaving. 

12. Synchronization solution (10 ml): 5 ml H_2_O, 3 ml 12 % NaClO and 2 ml 4 M NaOH^3^.

#### 1.3 Equipment

1. Stereomicroscope.

2. NIKON SMZ800 microscope equipped with a DS-Fi2 digital camera.

3. NIS Elements Documentation Imaging Software (Nikon, USA).

4. 96 well microtiter plates (rounded bottom).

5. 24 well microtiter plates (flat bottom).

6. 9 cm ø Petri plates with caps.

7. Thermomixer comfort (Eppendorf, Hamburg, Germany).

8. Pasteur pipettes, centrifuge tubes (15 ml and 50 ml) and Eppendorf tubes.

9. Incubator to culture *C. elegans* at 15 °C, 20 °C and 25 °C (Memmert, Schwabach, Germany).

### 2 Methods

#### 2.1 C. elegans culture and synchronization

*C. elegans* culture is maintained on Petri dishes with NGM at 20 °C. The plates are seeded with a lawn of OP50, an uracil-auxotroph *E. coli* strain (Brenner, 1974^[2]^)^4^. In order to obtain an age-synchronized group, worms are treated with the described synchronization solution as previously described (Piechulek et al., 2020[16]). The eggs will develop into L4 larvae after approximately 44 h (Altun et al., 2021^[1]^)^5^.

#### 2.2 Cultivation in 96 well microtiter plates

1. Prepare the S-medium described as before (see 2.2). 

2. Weigh the respective number of 50 ml centrifuge tubes needed for the test and fill up with overnight cultured OP50.

3. Centrifuge the falcons for 10 min at 2000 rcf at 20 °C, discard the supernatant. Then, weigh the falcons again and calculate the OP50 pellets. 

4. Based on the mass of OP50 pellets, calculate the volume of S-medium needed for a bacteria concentration of 18 mg/ml and re-suspend the bacteria pellet in the respective volume of S-medium.

5. Wash the L4 larvae from the plates into 15 ml centrifuge tube using M9. After the worms have sunk to the bottom, remove the supernatant.

6. Wash the larvae three times with 15 ml M9 and centrifuge at 250 rcf for 90 sec.

7. Transfer the worms with a glass pipette, into the 50 ml falcons containing 18 mg/ml OP50 to reach an amount of 10-15 worms per 99 µl, approximately.

8. Check the number of worms under a microscope by pipetting a drop of 99 µl worm suspension onto a microscope slide^6^.

9. Leave the external rows of the 96 well microtiter plate free and fill with 150 - 200 µl H_2_O to avoid desiccation.

10. Pipette 99 µl into each well of a 96 well microtiter plate with a shortened tip using the multi-step pipette.

11. After half of the plate has been pipetted, invert the 50 ml falcon again to ensure the equal distribution of worms within the medium. The plates will be stored in the incubator at 20 °C.

12. At the late stage of L4, add 36 µl of 6.25 mM of 5-Fluoro-2'-deoxyuridine (FUdR) dissolved in H_2_O to the S-medium in the 96 well microtiter plates, shake the plates at 1000 rpm for 1 min and store them in an incubator at 20 °C^7^. This prevents worms from self-fertilizing and maintains an age-synchronous culture (Gandhi et al.,1980[10]; Petrascheck et al. 2007[15]; Piechulek et al., 2018[18]).

#### 2.3 Exposure to pollutants or other environmental factors

At the first day of adulthood, the worms are exposed to pollutants and/or non-chemical stressors.

Non-chemical stressor / temperature:

1. Add 15 µl H_2_O to every well.

2. The plates are shaken at 1000 rpm for 1 minute at 20 °C. 

3. The plates are then incubated at 15 °C, 20 °C and 25 °C in respective incubators^8^.

#### 2.4 Fitness test 

The worms are submitted to the thrashing assay after 24 hours. Yet, prior to recording, several points need to be considered:

1. Check the plates under the optical microscope and cross out those wells where OP50 is absent. These worms cannot be used for testing, as they have hungered and would distort further analyses.

2. Before recording, shake the 96 well microtiter plates for 1 min at 1000 rpm.

3. Pipette up and down the liquid medium - including worms - from a well of 96 well microtiter plate several times before transferring it onto 24 well microtiter plate. Transfer 120 µl from one well (96 well plate) onto the 24 well plate (flat bottom) to form a drop in the center of the well^6^.

4. Record 30 seconds videos of the worms. Worms are filmed with a NIKON SMZ800 microscope equipped with a DS-Fi2 digital camera. Videos are recorded with NIS Elements Documentation Imaging Software (Nikon, USA). The settings for the videos are as follows: 

The quality should be set to “fast”. The focus is set to “normal”. For the compression, use “MJPEG compression”. The zoom factor is “1”.

5. If necessary, the worms can be filmed in the 96 well plate. For this, use a zoom factor of “2”.

6. In total, 3 to 5 wells per treatment group should be filmed to record enough worms for statistical analysis. 

7. After testing, discard the 24 well microtiter plate and store the 96 well microtiter plate in the incubators of the respective exposure temperatures; either 15 °C, 20 °C or 25 °C.

8. Filming is repeated on the respective days of testing^7^. 

#### 2.5 Thrashing assay

Thrashes of worms are counted based on the recorded videos.

1. Count the thrashes of about 15 worms from each group. A single thrash is counted when the body bents to one side and then back to the initial posture. 

2. In order to count the thrashing more accurately, the speed of recorded videos can be reduced to 0.5x or 0.75x.

3. Do not count the thrashing of those worms if:

a. They are still in larval stages.

b. They are in blind spots of the camera.

c. They reach the edge of drop and stop thrashing.

d. They are starved or damaged, *e.g.* leakage of the intestine.

e. They are hindered in their movement by OP50 stuck to them.

4. Analyze the data with GraphPad Prism (GraphPad Software, San Diego, CA, US) or Origin (OriginLab®, 2021).

## Results

**Figure 1:**
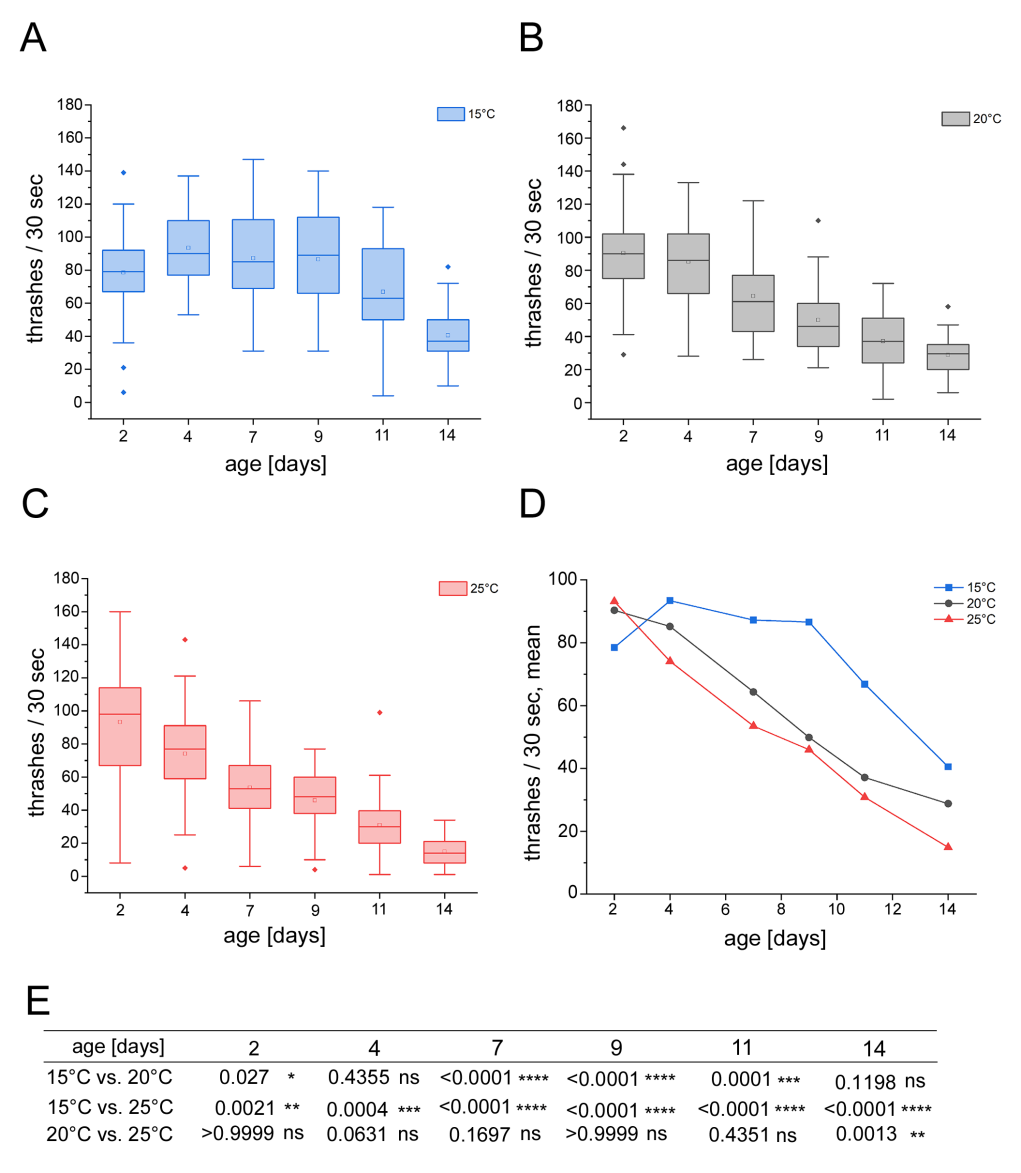
Age-resolved quantification of swimming fitness in adult hermaphrodite *C. elegans* cultivated at 15, 20 or 25 °C. Wild type *C. elegans* (N2) were cultured in 96 well microtiter plates in liquid S-complete medium containing live *E. coli* OP50. The worms were exposed to (A) 15 °C, (B), 20 °C and (C) 25 °C. The number of thrashes was counted by stereo microscopy and plotted against the age of adult worms from day 2 (young *C. elegans*) until day 14 (old-age *C. elegans*) in a boxplot diagram. The boxplots show the median, quantiles, outliers and mean values (small square). (D) The mean number of thrashes per 30 seconds (A-C) was plotted as a line chart. The color code for the different temperatures is presented on the right. Locomotion fitness was quantified by scoring the number of body bends per 30 seconds. Worms which did not display at least one thrashing movement were censored. (E) Significance (p) was compared between the exposure temperatures at different days (Kruskal-Wallis ANOVA with Dunn's post hoc test). All experiments were performed in triplicate. n ≥ 25 - 75. C, Celsius; d, days; ns, not significant

The prevalent locomotion of adult *C. elegans* in liquid is swimming that is characterized by a pattern of C-shaped undulations with high frequency and long wavelength (Cohen and Sanders, 2014[5]). To measure swimming fitness in relation with worm age and the non-chemical environmental factor temperature, worms were cultivated in liquid S-medium on 96 well microtiter plates between 2 and 14 days at temperatures of 15, 20 or 25 °C. For the fitness test, swimming thrashes were quantified. Worms were transferred to 24 well plates filled with liquid S-medium. Two-day-old adult *C. elegans *that were cultivated at 15 °C thrashed with a mean value of 78.5 times in 30 seconds (Figure 1A, D(Fig. 1)). Until an age of 9 days, this fitness increased to mean values between 93.4 (4-day-old) and 86.6 (9-day-old) thrashes. The thrashing frequency then declined in older worms (Figure 1A, D(Fig. 1)). In contrast, *C. elegans* cultivated at 20 or 25 °C showed an age-associated linear decrease in thrashing from the start until the end of the experiment, *e.g.* between 2 and 14 days (Figure 1B-D(Fig. 1)). When compared, 2-day-old worms held at 15 °C significantly thrashed less than their counterparts held at higher temperatures (Figure 1E(Fig. 1)). However, this switched in 4-day-old worms, and from an age of 7 to 14 days adult *C. elegans* that were raised at 15 °C significantly thrashed more than worms raised at higher temperatures. We observed that the '15 °C worms' kept the high level of swimming fitness until middle age (11-day-old) which then declined in older *C. elegans*.

In contrast, cultivation conditions of 20 or 25 °C induced a constant decline of the swimming fitness in young, middle-aged and old worms. This suggests that the enhanced swimming fitness observed in the '15 °C worms' is specific.

Temperature has been shown to modulate longevity in ectotherms and homeotherms, as it was demonstrated that reduction of the ambient temperature can increase life span (Conti et al., 2006[6]). In the ectotherm animal model *C. elegans* exposure of adult hermaphrodites to low-temperature prolongs life span via thermosensory neurons and the thermosensitive TRP channel TRPA-1 that acts upstream of the transcription factor daf-16/FOXO (Lee and Kenyon, 2009[14]; Zhang et al., 2015[28]). Here, we show that cultivation of adult *C. elegans* at the low temperature of 15 °C specifically enhances the neuromuscular phenotype of swimming fitness in young and middle-aged worms. As we do not yet understand the underlying molecular pathways, more investigation is required concerning the interactions between non-chemical environmental cues such as temperature and the genetic response in aging processes.

## Conclusions / Outlook

Here, we introduce an age-resolved fitness test in the nematode animal model *C. elegans* that quantifies the neuromuscular phenotype of swimming in young, middle-aged and old worms. In order to investigate a non-chemical environmental factor, we analyzed neuromuscular fitness in adult *C. elegans *that were cultivated at 15, 20 or 25 °C and provide a respective step-by-step protocol. Worms exposed to the higher temperatures showed a linear, age-related decrease of swimming activity from young to old age. In contrast, cultivation at 15 °C enhanced swimming fitness until day nine and postponed the age-related decline of the locomotion phenotype until the transition from middle to old age. Thus, we conclude that the lower cultivation temperature increases the health span of adult hermaphrodite *C. elegans*.

**Figure 2:**
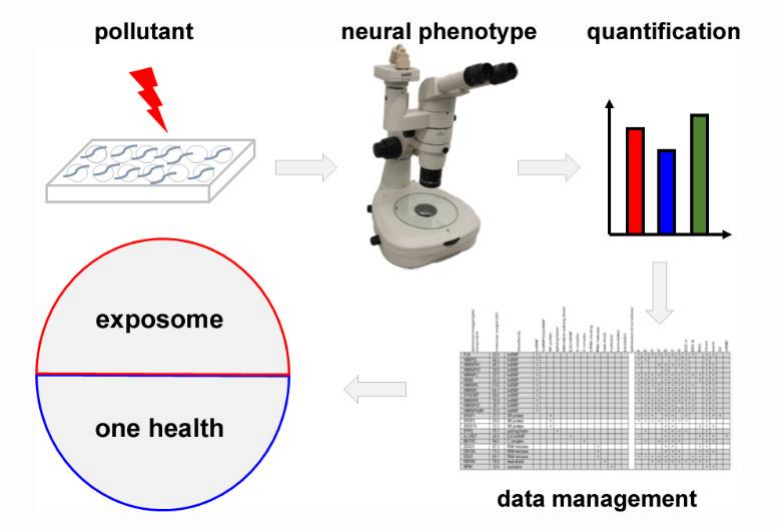
Workflow of life span-resolved fitness tests in the animal model *Caenorhabditis elegans* (schematic). Hermaphrodite *C. elegans* are exposed to pollutants and/or non-chemical stressors such as temperature during their entire adult life. Neural phenotypes are observed as biological response to toxicants by (video-)microscopy, quantified and analyzed by statistics (upper row). Current developments include automation, data collection and feeding into the concepts of the exposome and one health (bottom row).

We currently broaden the application of the presented microtiter well fitness test concerning: (i) comparative analyses of widely known and emerging neurotoxins, (ii) combination of neurotoxic pollutants and the non-chemical environmental factor ambient temperature, (iii) identification of vulnerable age-groups and their unbiased transcriptomic and proteomic profiling, (iv) automation of the phenotype quantification and (v) development of a capable data management (Figure 2(Fig. 2)). This tiered approach is designed to feed the exposome. With a current inventory of over 350,000 chemicals, including neurotoxins and their mixtures, it is evident that not all aspects of chemical pollution and the biological responses concerning the neural system can be characterized (Wang et al., 2020[26]). However, fast and cost-effective neurotoxicity testing in realistic target organisms represents an important tool. As a contaminated environment threatens the health of ecosystems and humans alike, the term of one health has been introduced. Consistent with this idea, the exposome concept comprises all environmental exposures across the life span of individuals and addresses chemical as well as non-chemical stressors in the environment (Wang et al., 2020[26]; Gao, 2021[11]). By feeding the data of age-resolved fitness tests in the nematode *C. elegans* into the exposome, the neuromuscular response of invertebrates that generally live in environmental sinks of anthropogenic pollutants contributes to the concept of one health.

## Notes

Indra Hering and Dang Tri Le contributed equally as first author.

^1^ Avoid damaging the surface of the NGM and spread *E. coli* to the edges of the plate to prevent *C. elegans* from crawling into the agar or off the plate, respectively.

^2^ Overnight cultures of *E. coli* OP50 grown in LB-Medium are used to make concentrated pellets of bacteria.

^3^ Be careful with NaOH and NaClO since they are corrosive.

^4^ Avoid starving of adult *C. elegans* hermaphrodites, because starved worms develop an internal hatch phenotype and eggs cannot be isolated anymore. Food should always be abundant.

^5^ It is recommended to monitor the development in every experiment. L4 larvae are recognized by a characteristic white triangle at the developing vulva.

^6^ Tips have been cut 2 mm - 4 mm from the head (shortened tips) to prevent damaging worms. 

^7^ The final concentration of FUdR in each well should be 1.5 mM FUdR.

^8^ Shake the plate every second day in long-term experiments to mix up the components in the wells.

## Declaration

### Acknowledgments

We thank the German Research Foundation DFG (Grant MI 486/10-1) for financial support, the Caenorhabditis Genetics Center (CGC, University of Minnesota, Minneapolis, USA) for providing *C. elegans* strains and members of the von Mikecz lab for critical discussions.

## References

[1] Altun ZF, Hall DH Handbook of C. elegans anatomy. WormAtlas.

[2] Brenner S (1974). The genetics of Caenorhabditis elegans. Genetics.

[3] Brewer JC, Olson AC, Collins KM, Koelle MR (2019). Serotonin and neuropeptides are both released by the HSN command neuron to initiate Caenorhabditis elegans egg laying. PLoS Genet.

[4] C. elegans Sequencing Consortium (1998). Genome sequence of the nematode C. elegans: a platform for investigating biology. Science.

[5] Cohen N, Sanders T (2014). Nematode locomotion: dissecting the neuronal-environmental loop. Curr Opin Neurobiol.

[6] Conti B, Sanchez-Alavez M, Winsky-Sommerer R, Morale MC, Lucero J, Brownell S (2006). Transgenic mice with a reduced core body temperature have an increased life span. Science.

[7] Corsi AK, Wightman B, Chalfie M (2015). A transparent window into biology: a primer on Caenorhabditis elegans. Genetics.

[8] Danovaro R, Fanelli E, Aguzzi J, Billett D, Carugati L, Corinaldesi C (2020). Ecological variables for developing a global deep-ocean monitoring and conservation strategy. Nat Ecol Evol.

[9] de Bono M, Maricq AV (2005). Neuronal substrates of complex behaviors in C. elegans. Annu Rev Neurosci.

[10] Gandhi S, Santelli J, Mitchell DH, Stiles JW, Sanadi DR (1980). A simple method for maintaining large, aging populations of Caenorhabditis elegans. Mech Ageing Dev.

[11] Gao P (2021). The exposome in the era of one health. Environ Sci Technol.

[12] International Human Genome Sequencing Consortium (2001). Initial sequencing and analysis of the human genome. Nature.

[13] Kaletta T, Hengartner MO (2006). Finding function in novel targets: C. elegans as a model organism. Nat Rev Drug Discov.

[14] Lee SJ, Kenyon C (2009). Regulation of the longevity response to temperature by thermosensory neurons in Caenorhabditis elegans. Curr Biol.

[15] Petrascheck M, Ye X, Buck LB (2007). An antidepressant that extends lifespan in adult Caenorhabditis elegans. Nature.

[16] Piechulek A, Berwanger L, Hemmerich P, von Mikecz A (2020). The nucleus of intestinal cells of the bacterivore nematode Caenorhabditis elegans as a sensitive sensor of environmental pollutants. Methods Mol Biol.

[17] Piechulek A, Berwanger LC, von Mikecz A (2019). Silica nanoparticles disrupt OPT-2/PEP-2-dependent trafficking of nutrient peptides in the intestinal epithelium. Nanotoxicology.

[18] Piechulek A, von Mikecz A (2019). Aging by pollutants: introducing the aging dose (AD)50. Environ Sci Eur.

[19] Piechulek A, von Mikecz A (2018). Life span-resolved nanotoxicology enables identification of age-associated neuromuscular vulnerabilities in the nematode Caenorhabditis elegans. Environ Pollut.

[20] Scharf A, Gührs KH, von Mikecz A (2016). Anti-amyloid compounds protect from silica nanoparticle-induced neurotoxicity in the nematode C. elegans. Nanotoxicology.

[21] Scharf A, Piechulek A, von Mikecz A (2013). Effect of nanoparticles on the biochemical and behavioral aging phenotype of the nematode Caenorhabditis elegans. ACS Nano.

[22] Solis GM, Petrascheck M (2011). Measuring Caenorhabditis elegans life span in 96 well microtiter plates. J Vis Exp.

[23] Stiernagle T, Hope IA (1999). Maintenance of C. elegans. C. elegans. A practical approach.

[24] von Mikecz A (2018). Lifetime eco-nanotoxicology in an adult organism: where and when is the invertebrate C. elegans vulnerable?. Environ Sci Nano.

[25] Wang Y, Nowack B (2018). Dynamic probabilistic material flow analysis of nano-SiO2, nano iron oxides, nano-CeO2, nano-Al2O3, and quantum dots in seven European regions. Environ Pollut.

[26] Wang Z, Walker GW, Muir DCG, Nagatani-Yoshida K (2020). Toward a global understanding of chemical pollution: a first comprehensive analysis of national and regional chemical inventories. Environ Sci Technol.

[27] Yuan B, McLachlan MS, Roos AM, Simon M, Strid A, de Wit CA (2021). Long-chain chlorinated paraffins have reached he arctic. Environ Sci Technol Lett.

[28] Zhang B, Xiao R, Ronan EA, He Y, Hsu AL, Liu J (2015). Environmental temperature differentially modulates C. elegans longevity through a thermosensitive TRP channel. Cell Rep.

[29] Zhang P, Carlsten C, Chaleckis R, Hanhineva K, Huang M, Isobe T (2021). Defining the scope of exposome studies and research needs from a multidisciplinary perspective. Environ Sci Technol Lett.

